# IoT Architecture-Based Mechanism for Digital Transmission of Key Aspects of the Enterprise

**DOI:** 10.1155/2022/3461850

**Published:** 2022-03-18

**Authors:** Cheng Chen

**Affiliations:** School of Economics and Management, Tongji University, Shanghai 200092, China

## Abstract

This paper provides an in-depth study and analysis of data transmission in key aspects of the enterprise using internet of things (IoT) technology. IoT technology can provide an effective solution to the integration of enterprise resources and efficiency improvement. If it can be properly introduced into the enterprise, it not only can effectively integrate the enterprise resources in the management but also can significantly improve the efficiency and thus further reduce its operating cost. This paper explores the management application strategy of IoT in the key aspects of digital transmission in enterprises. It can also increase productivity to address labour shortages. Most importantly, because of the internet of things technology, many emerging industries have been derived. In order to achieve this research objective, firstly, based on the in-depth study of the problems of digital transmission of key aspects of the enterprise, this paper evaluates and summarizes the supply chain, safety, efficiency, and quality problems that often occur in the past operation and management of the case enterprise and analyses the main reasons for the formation of the problems and then recognizes the necessity and feasibility of introducing IoT technology and management; secondly, based on the kernel of IoT and industrial IoT technology combined with the main modules of enterprise operation management, using the 5S field management method, we try to build a three-dimensional all-round electrical equipment enterprise IoT management application scheme design system; and finally according to the planning and design rules and management process, we focus on implementing digital transmission network application projects from the enterprise level combining key links of technology and management. This paper studies not only technical innovation but also a revolution in management. The research in this paper will provide a more feasible technical and management design scheme for the application of IoT in enterprises for industry reference.

## 1. Introduction

The role played by the new generation of technology products in human life is irreplaceable, and the former science fiction movie scenarios are being used in modern life one by one [1, 2]. According to a study by the Internet Data Center (IDC), by 2020, there will be 29.5 billion connected devices worldwide, creating an IoT market of $1.7 trillion. According to a study by McKinsey & Company, a world-class consultancy, the economic impact of the IoT will be worth $11.1 trillion to $13.9 trillion per year by 2025, with industrial applications worth $1.2 trillion to $3.7 trillion, making it the largest economic application of all [3]. Many countries have included IoT as an economic development priority in their national strategies. This shows that IoT is the future technology trend in human development, and its application not only can improve the quality of products and services and increase the source of customers but also can increase productivity to solve the labour shortage. Most importantly, many new industries have emerged because of IoT technology [4].

In recent years, the emergence of IoT technology has improved people's quality of life, and many countries have incorporated IoT into their national strategies for economic development, especially in manufacturing, which is predicted to be the largest economic application area in the future [5]. In the decade since its emergence to the present day, blockchain technology has grown rapidly into mature and practical information technology [6]. Blockchain can be thought of as a distributed ledger that is monitored and maintained by all participants through the network, thus ensuring the authenticity and immutability of information. At the same time, blockchain, as a “decentralized trust network,” can maximize the elimination of human intervention, and each node in the network does not need to know who the other is if it conforms to the consensus encryption mechanism to complete the operation and achieve distributed storage [7]. Blockchain's decentralization, immutability, openness, and transparency provide a new path for the development of digital transmission of key aspects of enterprises.

With IoT technology as the core, embedded development technology, wireless sensor network technology, and fault diagnosis technology are applied to realize the wireless monitoring and evaluation system of the pump group based on IoT [8]. The sensor is used to monitor the running state of the centrifugal pump set, and the running data of the centrifugal pump in the current state are collected and uploaded. The cloud server performs health assessment, fault diagnosis, and fault prediction on the current state of the centrifugal pump and gives corresponding treatment measures and treatment suggestions according to the system's evaluation of the current state of the centrifugal pump. This is conducive to the timely detection and troubleshooting of faults by the staff and prolongs the service life of the equipment.

## 2. Related Works

**Figure 1:**
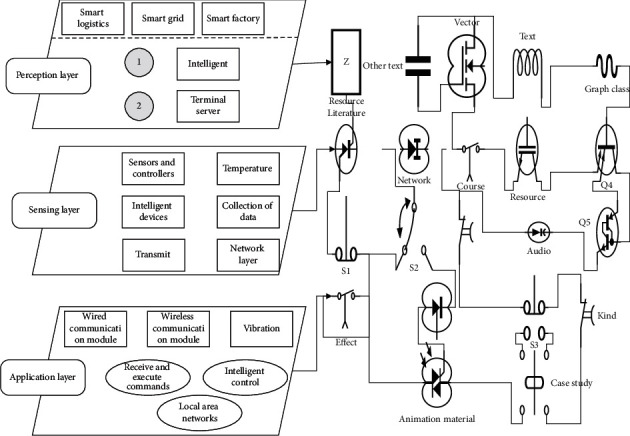
Simple architecture of industrial IoT.

The industrial internet of things (IoT) is an information technology based on the Internet, which integrates various sensors, intelligent terminals, and other advanced devices by various means to achieve the interconnection of everything [9]. As shown in Figure 1, the industrial IoT architecture can be roughly divided into three layers: the sensing layer, the network layer, and the application layer. The perception layer of industrial IoT consists of many sensors and controllers, mainly including various types of sensors such as vibration, pressure, temperature and humidity, RFID tags, QR codes, and various intelligent devices. The main task of the sensing layer is to realize the collection of data from various devices and transmit the collected data to the network layer, receive and execute the commands from the network layer, realize intelligent control, and realize a wireless monitoring and evaluation system for pump groups based on the internet of things. The sensing layer of industrial IoT consists of many sensors and controllers, mainly including various types of sensors such as vibration, pressure, temperature and humidity, RFID tags, QR codes, and various smart devices [10]. The main task of the sensing layer is to realize the collection of data from various devices, transmit the collected data to the network layer, and be able to receive and execute commands from the network layer and realize intelligent control. It is mainly responsible for the transmission of various types of information and is the link for the transmission of information throughout the industrial internet of things [11]. The application layer is mainly responsible for data storage, data processing, analysis and decision-making, and so on. It controls the perception layer to carry out corresponding actions and realize remote monitoring and control of industrial equipment to achieve the purpose of human-computer interaction in IoT.

From the perspective of the internet of things technology, its origin, development, and popularization in foreign countries started early, so the overall system technology supported by the internet of things technology applied in different directions and fields is also completer and more comprehensive. It is of great significance to give corresponding treatment measures and treatment suggestions, which will help staff find and eliminate faults in a timely manner, prolong the service life of equipment, and reduce the number of equipment downtimes. Internet of things technology is a multi-disciplinary, comprehensive technology system. It involves computers, with wireless communication, multinetwork interconnection, sensor collection, and integration technology [12]. Foreign scholars and even development teams belonging to companies have invested a lot in hardware technology support or software application and correlational and collaborative research, and some technologies are even in a forward-looking position in the world. This network is usually part of an ecosystem (ecosystem) that includes connectivity between things, applications, and data analysis. Although networks are not a prerequisite for internet systems, the value of the internet of things being realized has the potential to create a huge and global impact [13].

Concerning the impact of IoT on business operation and management, industrial IoT has indeed developed rapidly and vigorously in recent years, and production automation and process automation have improved quality and efficiency for many enterprises, in addition to which the return on investment has also been quickly obtained [14]. In this paper, we propose and study an enterprise key link digital transmission system based on the internet of things, which is supported by the three-layer structure of the internet of things as a big framework, involving node data collection, RFID access technology, data transmission module, and so on. The main components of the data are equipment status information, node water pressure at the collection end, and hardware control information; the system is intended to complete accurate data collection and smooth transmission at the same time to data information for additional processing, so that it can provide decision support for the overall system and the construction of the ontology-based monitoring and early warning system model. In terms of the data visualization user side based on the cloud data transit, a friendly interface, humanized interactive, and easy-to-operate program are developed for the web application side of the manager [15].

## 3. Enterprise Critical Link Digital Transmission System Model Setup

### 3.1. Mathematical Modelling

The overall supply chain traceability system architecture can be divided into three layers, mainly including the data access layer that obtains product data and status information and uploads them through the interface program; the data core layer that invokes smart contracts to complete the supply chain business logic and conducts blockchain data storage according to the received data information; and the application representation layer that includes the traceability query web page and WeChat traceability query applet [16]. It involves node data collection, RFID access technology, data transmission module, and so on. The main components of the data are equipment status information, collecting end node water pressure, and hardware control information. The system aims to complete accurate data collection and smooth transmission. Information undergoes additional processing so that it can provide decision support for the overall system.

The data access layer consists of various sensors, 4G modules for data upload, and the data upload app, which mainly completes the task of acquiring product data information and uploading it to the core layer. The ways to obtain the product data information include various types of sensor acquisition and scanning the product QR code using the data upload app, which has a unique QR code number, which is the unique identity of each food product, and the QR code number is generated by 32 bit MD5 encryption algorithm; the food product is photographed using the data upload app to obtain the actual photo. The type of data information acquired mainly includes the production or growth environment information collected by sensors, the product status information acquired by scanning the QR code, and the physical photos acquired by taking photos.

**Figure 2:**
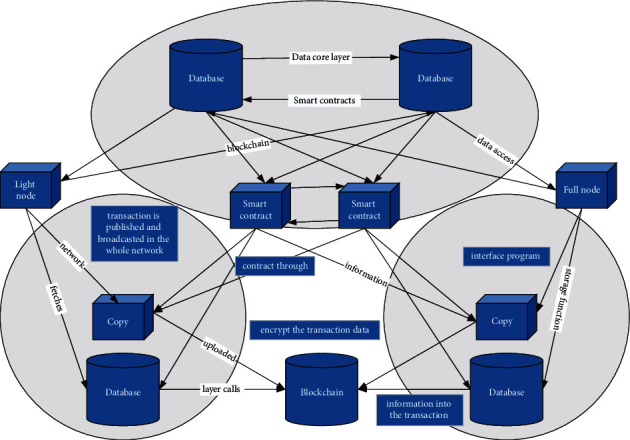
Data core layer architecture topology.

The main body of the data core layer is the blockchain network, aided by a generalized interface program and smart contracts, and the entire blockchain network consists of many blockchain nodes. The full-node server that fetches the uploaded information from the data access layer calls the storage function in the smart contract through the interface program to populate the information into the transaction data and encrypt the transaction data; then the transaction is published and broadcasted in the whole network; and then the transaction is packaged into a block and broadcasted to the whole blockchain network by the consensus algorithm verification of other nodes, and the block is added to the blockchain as the latest block after the completion of the verification.  Figure 2 represents the data storage process using the smart contract approach.

The user is registered through the user management module at the end of the web page and must fill in the required fields; the whole process is monitored by the administrator; the administrator account is assigned by the senior level of the system and does not have to be registered; and the new user information generated by the module will be stored to the cloud centre data [17].

Administrator users can obtain information through two-way data transmission with the database, then monitor different types and locations of node equipment, alarm equipment, and so on, and can view the status of the equipment and the adjustment of operable nodes. When a device fails, the device administrator can view the failed device without showing the cause of the device failure to the field and then request maintenance on the failed device. It mainly includes the data access layer that obtains product data and status information and uploads it through the interface program; the data core layer invokes smart contracts to complete the supply chain business logic, stores blockchain data according to the received data information, and includes the traceability query web page and the application presentation layer of the WeChat traceability query applet. After the approval is completed, the system will feed back the approval result. After the approval, the relevant technicians will be arranged to carry out maintenance and repair and upload the data records to the database for viewing after completion.

(1) $$\begin{array}{c} PR_{T}-W+C+R_{D}>0. \end{array}$$

This paper will be based on the modern production management concept and adopt the basic idea of joint optimization of management tools and technology, based on the characteristics of motor production in the information technology environment, for the current production operation of the Shanghai Electric Machinery Factory to carry out an in-depth study of the existing problems, based on which an improvement program is proposed: The design focus of this Internet of Things application scheme is the combination of production management methods and Internet of Things technology; this theoretical framework helps promote the deeper application of traditional production management methods and the mechanism of reuse in the introduction of IoT. The overall idea is to combine the theories of IoT and production management with the main problems of production management and operation management of Shanghai Electric Factory. The solution starts from the needs of the production operation of the enterprise, and then the architecture of the system is carried out.

**Figure 3:**
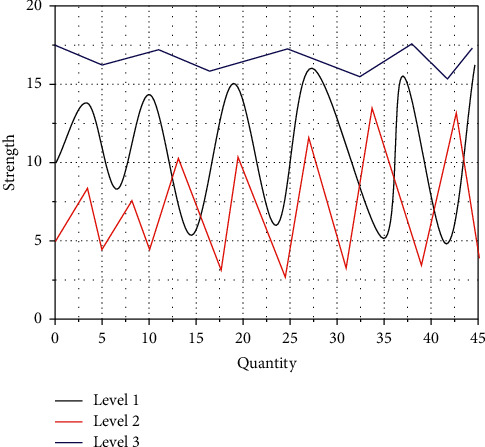
Fluctuation of the adaptive alarm valve.

Another constraint on efficiency is site management, and 5S is a powerful tool. Companies can improve efficiency in the production process while employees can work safely, healthily, and comfortably, and at the same time, companies want to reduce costs; they must start with manufacturing costs that have a high-cost structure. The product cost of the company accounts for 80% of the total cost of the company, while the manufacturing cost (direct labour and equipment improvement) accounts for about 20%, so this study will focus on the improvement of productivity through 5S to do research. In the combination with the internet of things (IoT), the main performance is that it can be supplemented with 5S management while using IoT. The full-node server that gets the information uploaded by the data access layer calls the storage function in the smart contract through the interface program to fill the information into the transaction data and encrypt the transaction data and then broadcast the transaction in the entire network, and then pass the data of other nodes. After the consensus algorithm is verified and the transaction is packaged into blocks, it is broadcasted to the entire blockchain network. In the process of 5S management, IoT technology is actively used, such as through the completion of RFID reading and writing method tests, business process combing, and so on, to provide technical means for on-site management. Simple simulation once, the fluctuation of the adaptive alarm valve is shown in Figure 3.

Finally, through all the data collection devices on-site, the production quantity, yield rate, work-in-process distribution, equipment operation, personnel efficiency, equipment efficiency, and quality inspection results, and other real-time on-site consultation is fed back to the ERP system. Therefore, to grasp the production status of the manufacturing site, information collectors, such as handheld barcode machines or fixed barcode machines, are usually installed at each production line input station to collect various production information, operation status, environmental data, quality data, and so on through these barcode machines and then store this information in the database of the MES system. You can view device status and adjust operational nodes. When a device fails, the device administrator can view the faulty device without showing the cause of the device failure to the site and then apply for maintenance of the faulty device.

(2) $$\begin{array}{c} C=\frac{E_{\min}\left|x\left(t\right)\right|}{\sum_{t=1}^{\sqrt{n^{3}}}x\left(t^{2}\right)}. \end{array}$$

### 3.2. Design of Digital Transmission Systems for Key Aspects of the Enterprise

The cost and energy consumption of LoRa wireless communication technology are lower than those of NB-IoT wireless communication technology, and in the actual industrial application, the branch units are often located in remote places, so the NB-IoT technology may not be covered by the operator, so LoRa technology is used as the communication method for wireless monitoring in this paper [18].

(3) $$\begin{array}{c} \delta =\frac{g}{d},\\ d=\sum_{i,j=1}^{N}\max d_{ij}. \end{array}$$

**Figure 4:**
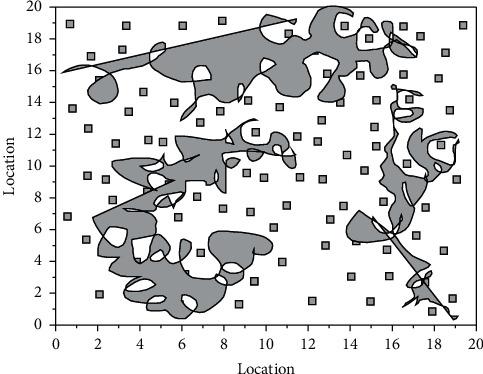
Distribution of nodes.

In MATLAB 2015a software, consider a random deployment of 50 sensor nodes in a fixed square area with a side length of 100 m. These nodes are static and do not have information about their location. A mobile NB-IoT node joins this cluster and is first at position 1 (0, 0), then moves to position 2 (33, 0), position 3 (67, 0), position 4 (100, 0), position 5 (100, 33), position 6 (100, 67), position 7 (100, 100), position 8 (67, 100), position 9 (33, 100), position 10 (0, 100), position 11 (0, 67), and position 12 (0, 33). The single- or multi-hop information from all nodes in the region is received at each location stop, and this one with the smallest value of node hops passed from each node is recorded and noted as the shortest path. The node distribution is shown in Figure 4.

In Figure 4, it is the location of the mobile cluster head localization stay of NB-IoT, and ○ is the node whose location is unknown and needs to perform the localization calculation. The minimum value of the two-two combined hop count sum between any node to the 12 anchor locations is used as the shortest path between the two locations by 12 times measured hop count calculation. The application scheme of the internet of things is proposed, which is composed of multiple modules. The solution starts from the needs of the production and operation of the enterprise and then carries out the architecture of the system. An estimated jump distance value is then calculated by equation. After calculation, the node positioning error accuracy is 32.81% on average for 20 times, which achieves the position positioning function of the unknown node, but the error degree is still high. Through the analysis of the influence of related factors and parameters on the accuracy, a better combination of positioning-related parameters is obtained for more accurate positioning.

The importance of smart contract security is increasingly recognized with the occurrence of well-known blockchain projects such as the DAO and Parity multi-signature wallets whose market capitalization evaporated overnight due to contract vulnerabilities that were attacked and caused huge losses. Formal verification is a security audit method for testing the security of smart contracts [19, 20]. Formal verification is a method that uses mathematical models to logically test hardware or software and predict results. For smart contracts, performing formal verification provides good assurance that the problems described in the previous section will not occur during the execution of the smart contract code, maximizing the security and reliability of the entire system.

## 4. Results and Analysis

### 4.1. Mathematical Model Results

In recent years, with the continuous development of Internet of Things technology, automatic sensing technology for tank level monitoring has achieved comprehensive development. After prudent research, it is believed that such aspects as an automatic level metering system, oil leakage and oil shortage real-time monitoring system, and so on can be effectively integrated with the help of intelligent internet of things platform while realizing the real-time transmission of oil tank information to enhance the overall monitoring and management of the equipment CNC bus level: (1) system advantages: each external site storage tank area has a wide range of distribution space, monitoring points, wiring complex, and other objective disadvantages and (2) real-time management: for enterprise decision-making, IoT technology allows more real-time correct management can be achieved; site managers can view real-time production progress, equipment status, material inventory status, and other information through the central monitoring platform to make appropriate decisions and arrangements to improve production efficiency. At the same time, through automated data collection, production data can be collected more completely, and through statistical analysis, abnormal quality conditions can be detected early, and product yields can be improved, and costs reduced. For workers, IoT technology replaces tedious and labour-intensive actions, such as meter reading and routine inspection—allowing more time for higher-value work, such as production data analysis, production efficiency improvement, equipment maintenance, and so on and allowing their expertise to be utilized.

(4) $$\begin{array}{c} P\left[X\left(t_{n}\right)=X\left(t_{n+1}\right)\right]. \end{array}$$

If *P*_*r*_+*W* − *C*+*R*_*D*_ − *L* > 0, then *x* = 1 is the intelligent in WSNs the unique evolutionary stable state ESS of the node evolutionary game. In this communication setting, we set the value *P* to a constant. By increasing *P*_*r*_ or *W* or decreasing *C*, *R*_*D*_, or *L* in equation, we can satisfy the condition that will force nodes to avoid choosing the no-forwarding strategy. As the value *W* increases (regardless of which policy the node chooses in the initial state), all nodes will eventually choose the forwarding policy, and the system will reach a steady state. Under normal circumstances, the transmission rate can only reach about 37.5 kbps, so LoRa communication technology is suitable for wireless monitoring occasions with many centrifugal pumps and a wide distribution range that do not require high communication speed.

**Figure 5:**
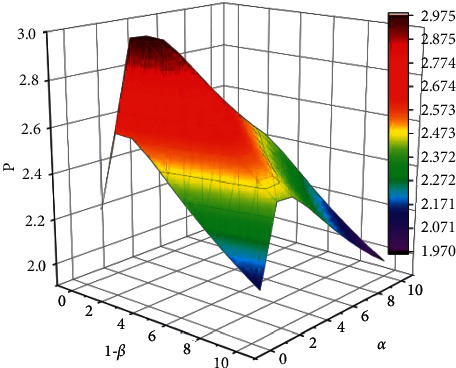
Plot of the variation between *α*, 1 + *β*, and *R*.

When a node is in a busy (backlogged) communication environment and the node selects the forwarding policy too often, it wastes the node's time and effort. Therefore, in this case, it is more beneficial to choose the no-forwarding policy. We give the incentive *r* for nodes choosing the no-forwarding policy. *R* is the average probability of packet forwarding, *R*=1 − *β*. If *α* or 1 − *β* decreases, *R* increases, and the positive correlation between *α*, 1+*β*, and *R* varies as shown in Figure 5.

There are various ways to increase the probability of packet forwarding in smart nodes in WSNs, such as increasing the bandwidth or frequency, improving hardware or transmission protocol algorithms, and so on. By increasing the R-value, *P*_*r*_+*W* − *C*+*R*_*D*_ − *L* tends to 0; then, nodes will increasingly choose the forwarding policy and the WSNs will reach the steady-state faster. In this case, if we increase *P*, it will not affect the final evolutionary steady state but will increase the speed of convergence to the evolutionary steady state. Conversely, if the value of *P* increases, it is difficult to satisfy the condition of Theorem 3 that *P*_*r*_+*W* − *C*+*R*_*D*_ − *L* < 0, and then the WSNs will slowly reach the steady state. In this case, if we increase *r*, it will not affect the final evolutionary steady state but will reduce the speed of convergence to the evolutionary steady state. The company's product cost accounts for 80% of the company's total cost, while the manufacturing cost (direct labour and equipment improvement) accounts for about 20%, so this research will focus on improving production efficiency through 5*S*.

We set the parameters to satisfy the conditions of the theorem, as shown in Table 1. The experimental results are shown in Figures 4 and 5, from which we can observe the trend of the evolution curve of the node states in the smart nodes in WSNs.

There are various ways to combine NB-IoT technology with WSNs; one of the options is to use NB-IoT nodes and WSNs nodes in a hybrid network according to their characteristics, playing the characteristics of traditional WSNs nodes such as random deployment, flexible networking, and low price and NB-IoT's wireless wide-area access, low efficacy and long life, and strong penetration capability. NB-IoT and WSNs hybrid network with aggregation/cluster head nodes and common sensing nodes. Common sensing nodes are the common number of sensor nodes that are randomly distributed within the monitoring area. We do not have high requirements for the data processing capability, storage capability, and communication capability of such nodes, and they are often composed of inexpensive miniature sensors whose function is to be responsible for data collection and data transmission. Aggregation/cluster head nodes for data aggregation, which are much more capable than ordinary sensor nodes, and their impact on the functional implementation of the overall WSNs is also significant. The sensor nodes send the collected data to the near-neighbour sensors, using a multi-hop transmission, and the data is finally aggregated to the aggregation node.

Wireless sensor nodes in different hierarchical positions and serving different functions have different node degrees. Sensor nodes with smaller node degrees have a single function, simple security mechanisms associated with them, and a higher probability of being controlled by viral malicious programs. Through the handheld barcode machine or fixed barcode machine, the production data, operating status, environmental data, quality data, and so on are collected, and then these data are stored in the database of the MES system. In contrast, sensor nodes with larger node degrees are fully considered in the network planning and design to be configured with system access rights restrictions, security firewalls, and other mechanisms so that their ability to cope with the prevention of attacks against cracking is enhanced, and the probability of being breached is reduced.

(5) $$\begin{array}{c} \xi \left(K\right)=\frac{m}{d\left(\kappa \right)}. \end{array}$$

### 4.2. Enterprise Key Link Digital Transmission System Model Simulation Experiments

To deploy a computer as a blockchain node, we need to install node.js, the Ethereum client, the git distributed version control system, and also the Truffle compiler for smart contract writing, including Solidity, Web3.js, and Solidity development framework. Environment variables are configured after installing Node.js, and the rest of the environment variables will be configured automatically after the software is installed. In a Linux environment, use apt-get and nm. Command in a terminal window to download and install the above software to complete the preparation work before setting up the environment.

**Figure 6:**
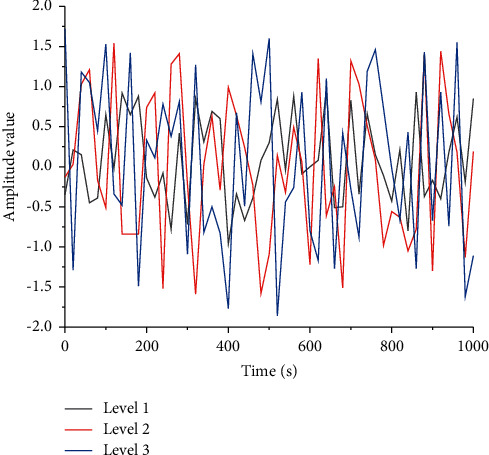
Fault time-domain data waveform.

Since EtherChannel is based on accounts to complete operations, after opening a blockchain node, the first step is to create a new account and enter personal. Click on the new account on the command line to create a new account, while EtherChannel will prompt to set a password. At this point, the account is locked and will fail when deploying and invoking smart contracts, so you need to use it personally. Since the recap set up when opening the blockchain allows access to the personal family of functions, the account can be automatically unlocked by writing this function to the Node.js interface. Before a smart contract can be called using the interface program, it needs to be compiled and deployed on the blockchain. The deployment of a smart contract on the Ethereum blockchain is done in the form of a published transaction, which is stored in the data field of the transaction. All blockchain nodes receive the transaction and perform signature verification and consensus algorithm to reach an agreement and achieve the storage of the contract on the chain. When a failure occurs in one part of the system, the system reacts and records it first, and the detailed test results of one cycle are shown in Figure 6.

By chance, we came across an AR game for preschoolers: the mobile app software scans a card with an animal pattern on it, and a three-dimensional image of the animal on the card appears on the phone with animal sounds, which helps children learn. This game uses card patterns as a medium to enhance human understanding and memory with three-dimensional images and sounds. This game has inspired us. Instead of card pattern information, QR code information or RFID information can be used in the workshop; three-dimensional images of animals can be replaced with various three-dimensional images of parts; and sound information can be presented in the form of explanations in drawings. The 2D code or RFID on-site can record various information such as the model number of the parts, their location, and their position in the finished product. The transmission distance of the signal in the open area can reach 10 km; the transmission distance in the town can reach 1∼2 km; and the maximum transmission rate can reach 250 Kbps. Because there are more young workers in the workshop, most of them like to play games; the way of learning through mobile phones is easily accepted; and the three-dimensional model pattern display is far easier to be understood than explaining with drawings. Through the mobile phone can also make the content of AR learning competition software to enhance learning initiative; in addition, through the two-dimensional code mark or RFID each work step into the system, you can trace the completed work and incomplete work, to achieve the guidance function of the work task, through the mandatory process of the production process and process control, to avoid errors in the work.

**Figure 7:**
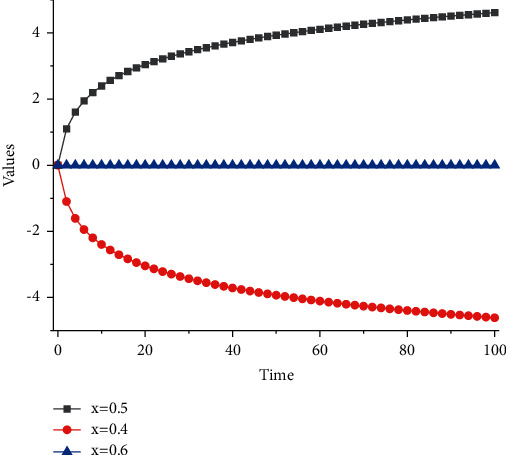
Schematic diagram of the evolution of packet forwarding curve denoted by *W*.

When the rate at which nodes choose a forwarding strategy in the initial state is 1%, reaching the stabilization point *χ*2^*∗*^ = 1 requires about 110 games, but if *W* = 4, reaching the same stabilization point requires only about 40 games. Satisfying the conditions of the theorem, the larger the cooperative incentive, the faster the intelligent nodes in WSNs reach the equilibrium point. When the rate of nodes choosing the forwarding strategy in the initial state is 99%, it takes more than 100 games to reach the stabilization point *χ*1^*∗*^ = 0 if *W* = 3, but only more than 80 games to reach the same stabilization point if *W* = 4. Under the condition that the theorem is satisfied, the gain of nodes that choose the forwarding strategy is smaller than the gain of nodes that do not choose the forwarding strategy. However, the cooperation incentive promotes cooperation among nodes to forward packets to each other, thus reducing the rate at which nodes reach the evolutionary steady state of intelligent nodes in WSNs, and the dynamic trend is shown in Figure 7.

ESP-12F has three working modes: STA, AP, and STA + AP hybrid mode. From the viewpoint of the usage requirements of this system, since the monitored temperature and humidity data, voltage, and related information need to be transmitted to the cloud platform, the system must be able to connect with the router connected to the external network to realize data exchange, so the main working mode should be set in STA mode to access the Wi-Fi network established by the hotspot already connected to the external network to realize the functions such as data upload.

The platform needs to be modularized to facilitate future expansion. Because the functions developed so far are only a very small part of the functions that the IoT can achieve and because the IoT technology is developing rapidly, it is very difficult to expand the platform later if it is not designed in a modular way, so it must be developed using a modular design. The platform must be a data presentation centre. As a platform software for industrial IoT, data presentation is the most basic function. After the data read from the sensing end are transmitted to the data collection end through the network bus, a large amount of data will be deposited in the storage; the platform needs to display two aspects of data; on the one hand, the currently collected data is displayed instantly so that employees can be informed of the current working status of the equipment; and on the other hand, historical data should also be displayed so that it can be compared with the best working status.

## 5. Conclusion

With the gradual launch of the 5G network, the new generation of the mobile communication network has more support and more openness for the connection of things, and the mobile communication network will play a greater role in promoting the development of IoT technology. The integration and development of IoT in various industries face a broader application prospect. Information technology, including the internet of things, is a technology for electrical equipment companies to connect customers, suppliers, and production processes; to overcome the information and communication between production equipment, so that production equipment can pass information to each other; to establish a key link between customers and suppliers through the internet of things; through the introduction of smart equipment and careful calculation of customer requirements, the production process is simplified and costs are reduced; the calculated information will be passed to production equipment and suppliers by introducing intelligent devices. We can finely calculate customer requirements; transfer the calculated information to production equipment and suppliers; achieve precise production; collect data affecting the production process; analyse, evaluate, and mine the data; and then establish a prediction model to make production equipment self-aware, self-memory, self-cognition, self-decision, and self-reconstruction. In the future, companies can use this model to produce different customized products and the lowest production cost.

## Figures and Tables

**Table 1:** Experimental data.

Group	*P*	*R*_*T*_	*R*_*D*_	*C*	*L*	*W*
1	0.424	0.81	0.905	0.693	0.959	0.783
2	0.639	0.611	0.751	0.567	0.479	0.647
3	0.966	0.701	0.568	0.679	0.414	0.849
4	0.889	0.485	0.916	0.755	0.941	0.848
5	0.982	0.745	0.629	0.623	0.748	0.476

## Data Availability

The data used to support the findings of this study are available from the author upon request.
